# High frequency of sexually transmitted infections in patients with precancerous cervical lesions in Brazil

**DOI:** 10.3389/fpubh.2025.1480959

**Published:** 2025-06-24

**Authors:** Kerellyn Follador, Leticia Viçosa Pires, Ana Paula Zanella Corbellini, Jefferson Henrique Zwir Poli, Rosilene Jara Reis, Mariana da Silveira Suñé, Priscila Lamb Wink, Giovana Cabrera, Giovana Fontes Rosin, Fernanda Uratani, Régis Kreitchmann, Helena Martins Mansur, Alessandro Comaru Pasqualotto

**Affiliations:** ^1^Santa Casa de Misericórdia de Porto Alegre, Department of Oncogynecological Surgery, Porto Alegre, Rio Grande do Sul, Brazil; ^2^Post-graduation Program in Pathology (UFCSPA), Porto Alegre, Brazil; ^3^Federal University of Health Sciences of Porto Alegre (UFCSPA), Department of Gynecology and Obstetrics, Rio Grande do Sul, Brazil

**Keywords:** sexually transmitted infections, human papillomavirus, Chlamydia, ureaplasma urealyticum, cervical intraepithelial neoplasia, cervical cancer

## Abstract

**Introduction:**

Cervical cancer is strongly associated with persistent human papillomavirus (HPV) infection, the most common sexually transmitted infection (STI) worldwide. While most infections are cleared naturally, co-infections with non-HPV STIs may contribute to HPV persistence and disease progression. Unlike cervical cancer, which has a national screening program in Brazil, STI screening remains unstructured, with prevalence varying across regions.

**Objective:**

To evaluate the prevalence of HPV co-infections with *Chlamydia trachomatis, Mycoplasma hominis, Mycoplasma genitalium, Neisseria gonorrhoeae, Ureaplasma urealyticum*, and *Trichomonas vaginalis* in patients diagnosed with cervical intraepithelial neoplasia (CIN) in Porto Alegre, Brazil. Methods: This cross-sectional study included patients with histologically confirmed precancerous cervical lesions attending a referral outpatient clinic. Between October 2022 and December 2023, 159 patients were enrolled and screened for the presence of non-HPV STI co-infections through cervical secretion DNA-qPCR testing.

**Results:**

Most (64.8%) participants were diagnosed with CIN II or III. Among all patients analyzed, nearly 60% had at least one non-HPV STI co-infection associated with low- or high-grade cervical lesions. The most prevalent pathogen was *U. urealyticum* (44%), followed by *M. hominis* (16.3%) and *C. trachomatis* (10.1%).

**Conclusion:**

A high prevalence of non-HPV STI co-infections was observed in asymptomatic women with CIN, particularly *U. urealyticum,* which has been identified as a potential cofactor in HPV-related carcinogenesis. Our findings contribute to the growing body of national and international literature supporting the need for integrating STI screening into cervical cancer prevention strategies for sexually active women in Brazil.

## 1 Introduction

Cervical cancer is the fourth leading gynecological malignancy worldwide and the fourth main cause of cancer-related mortality, with the highest incidence occurring in low- and middle-income countries (LMICs) (1, 2). In Brazil, cervical cancer ranks as the third leading cause of cancer-related deaths among women (1, 3).

Human papillomavirus (HPV) infection, which is highly prevalent—approximately 80% of women will contract HPV at some point in their lives—is strongly associated with cervical cancer. According to the World Health Organization (WHO), HPV infection currently affects over 290 million women worldwide (1, 4, 5). Latin America exhibits higher HPV prevalence rates compared to other regions (6). HPV is classified based on its oncogenic potential into low-risk types (which cause genital warts) and high-risk types (which are oncogenic and responsible for nearly 99% of precancerous cervical lesions, also known as cervical intraepithelial neoplasia – CIN, as well as cervical carcinoma) (7, 8). Among high-risk HPVs, types 16 and 18 are implicated in 70% of cervical cancer cases, while types 31, 33, 39, 45, 51, 56, 58, 59, and contribute to an additional 25% of cases (7).

However, not all women infected with high-risk HPV develop cervical cancer (7, 9). For carcinogenesis to occur, HPV persistence is essential, allowing progression to dysplasia, which, if followed by invasion, may result in malignant transformation. In more than 60% of cases, the infection resolves spontaneously within 12 months, and after 24 months, over 90% of infections either clear completely or remain in a latent state (7, 8, 10, 11). Even in cases of high-grade CIN (CIN II and III), many lesions regress naturally over time; however, 5 and 12% of CIN II and CIN III cases, respectively, may progress to invasive carcinoma (12).

Several behavioral factors are associated with HPV persistence, including age, immunosuppression, early sexual debut, multiple sexual partners, multiparity, low socioeconomic status, and smoking. Additionally, co-infections with other sexually transmitted infections (STIs) have been increasingly recognized as significant cofactors in HPV-related carcinogenesis. These STIs may contribute by causing chronic inflammation, microabrasions, and microtrauma in the cervical epithelium, increasing free radical production, impairing cellular immunity, and promoting angiogenesis (8, 13, 14).

Epidemiological studies have demonstrated an association between HPV infection, CIN, cervical cancer, and non-HPV STIs; however, the pathogenesis of this interaction remains poorly understood (6, 9, 15, 16).

It is estimated that more than 1 million treatable STIs are acquired worldwide each day, resulting in approximately 374 million new infections annually caused by one of the following four pathogens: *Chlamydia trachomatis, Neisseria gonorrhoeae, Treponema pallidum,* and *Trichomonas vaginalis* (1, 2, 17). Despite this high burden, the true incidence of these infections is likely underestimated, as most affected individuals remain asymptomatic (4, 17, 18).

In addition to these common STIs, co-infection with *Mycoplasmataceae* family pathogens, including *Mycoplasma hominis, Mycoplasma genitalium*, and *Ureaplasma urealyticum*, has been identified as a potential trigger for cellular alterations. These bacteria have been associated with HPV persistence and have been shown to induce chromosomal abnormalities and translocations *in vitro* (15, 19, 20).

The Brazilian Ministry of Health guidelines recommend cervical cancer screening via cytology every 3 years for all women aged 25 to 64 who have been sexually active (21). However, screening coverage remains suboptimal, with less than 30% of eligible women undergoing regular testing. Additionally, mortality rates have not declined significantly, and 60% of cervical cancer cases are still diagnosed at advanced stages (stage II or higher) (13).

Brazil has yet to implement a national molecular screening program for cervical HPV infection, and the current screening system remains opportunistic rather than organized. There is no active patient outreach, meaning that testing is infrequent, irregular, and often performed outside the recommended age range. Furthermore, other STIs are not included in the National STD/AIDS Program, with only HIV, syphilis, and hepatitis being subject to mandatory notification (10, 13, 22).

Given Brazil’s vast territorial extension and sociocultural diversity, access to primary healthcare services remains a challenge for many women, and health records in some regions are incomplete. Several epidemiological studies on HPV, CIN, and STIs have been conducted in different regions of the country, highlighting significant variations in population characteristics (16, 23).

The present study aims to evaluate the prevalence of non-HPV STI co-infections in asymptomatic women diagnosed with CIN in Rio Grande do Sul, the southernmost state of Brazil. Additionally, investigating the association of these infections with other risk factors and the severity of premalignant lesions may contribute to the existing body of national epidemiological data and help guide future cervical cancer prevention strategies in Brazil.

## 2 Materials and methods

Between October 2022 and December 2023, 159 patients were selected from the Reference Center for Lower Female Genital Tract Pathology at Hospital Santa Casa de Porto Alegre, affiliated with the Federal University of Health Sciences of Porto Alegre (UFCSPA). Patients were referred from primary care centers due to abnormal cervical cytology results for further colposcopy, biopsy, and treatment if necessary.

The sample size was calculated based on the quarterly number of patients attended at the center, aiming for a proportion of 16.5% (ranging from 6% to 27%), considering the mean prevalence of the most frequent non-HPV STIs reported in six previous studies. A 95% confidence interval (*p* < 0.05) and an absolute tolerated error of 5% were assumed.

Patient data, including age, self-reported race, risk factors (age at sexual debut, number of sexual partners, smoking, immunosuppression, educational level, socioeconomic status), and initial diagnosis, were extracted from medical records using an internally developed data collection tool. Patients were excluded if they were pregnant, had undergone a hysterectomy, chemotherapy, or radiotherapy, lacked histopathological confirmation of a premalignant cervical lesion, or presented symptoms suggestive of an STI, such as lower abdominal pain, abnormal uterine bleeding, or cervical discharge indicative of cervicitis.

Endocervical samples were collected using cotton swabs and analyzed via real-time polymerase chain reaction (qPCR) to detect *C. trachomatis, N. gonorrhoeae, T. vaginalis, M. hominis, M. genitalium,* and *U. urealyticum.* Samples were stored in CellPreserv medium (a methanol-based fixative solution, Kolplast®) and processed within 48 h before being discarded.

For molecular analyses (in house qPCR testing), primers and probes were obtained from Thermo Fisher Scientific (Massachusetts, United States). Each qPCR reaction contained 5 μL of extracted DNA, 10 μL of reaction mix with 1X GoTaq Probe qPCR MasterMix (Promega), 0.52 μM CXR Reference Dye (Promega), 0.5X primers and probes, and ultrapure water. The cycling conditions included an initial denaturation step at 95°C for 2 min, followed by 40 cycles of 15 s at 95°C and 60 s at 55°C.

Data was analyzed using SPSS v.22.0. Categorical variables were summarized as absolute frequencies and percentages, while quantitative variables were described using mean and standard deviation and compared using Fisher’s exact test. Quantitative data was compared using Student’s T test or Mann–Whitney test, depending on data distribution, with statistical significance *p* value of ≤0.05.

The study was approved by the Research Ethics Committee of Hospital Santa Casa de Porto Alegre (CAAE 60713522.7.0000.5335). All participants provided written informed consent before enrollment.

## 3 Results

**Figure 1:**
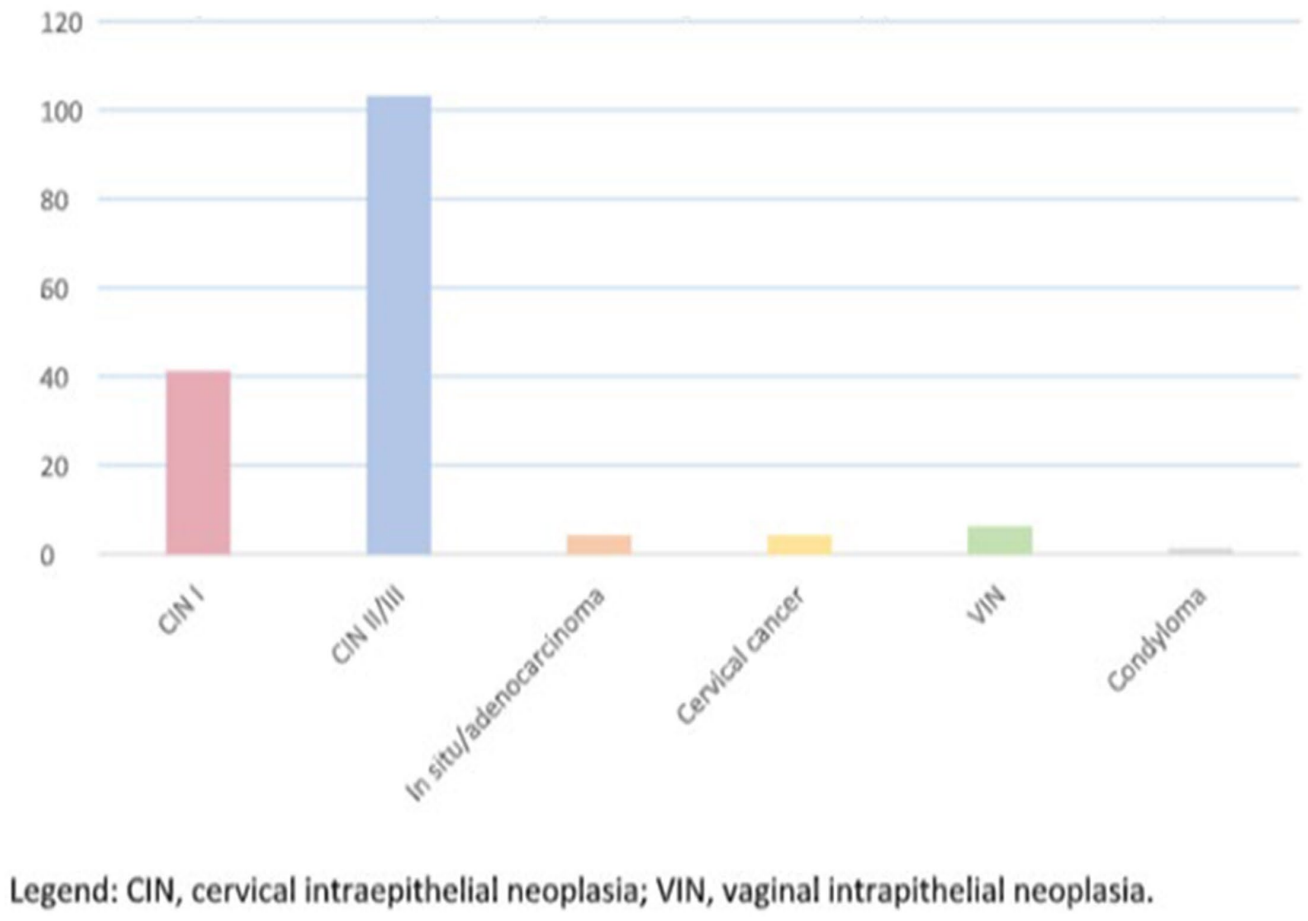
Main histopathological findings in the study (in absolute numbers). CIN, cervical intraepithelial neoplasia; VIN, vaginal intraepithelial neoplasia.

Among the 159 patients included in the study, 41 (25.8%) had low-grade cervical intraepithelial neoplasia (CIN I), while 103 (64.8%) had high-grade lesions (CIN II/III). Additionally, 8 patients (3.8%) were diagnosed with vaginal intraepithelial neoplasia (VIN), 4 (2.5%) had squamous cell carcinoma, 4 (2.5%) had adenocarcinoma *in situ,* and 1 patient (0.6%) was diagnosed with condylomatosis (Figure 1).

Regarding epidemiological data, more than 40% of the patients were from the capital city Porto Alegre, while the remainder came from other regions of Rio Grande do Sul. Smoking was reported by 24.5% of patients, and in the VIN group, the proportion of smokers and non-smokers was equal. Socioeconomic status analysis revealed that 74.8% of patients had a household income of up to two minimum wages, and only two patients reported earning more than five minimum wages. Regarding self-reported ethnicity, 69.8% identified as white, 17.6% as mixed-race (pardo), 10.7% as black, and 1.9% as Asian.

**Figure 2:**
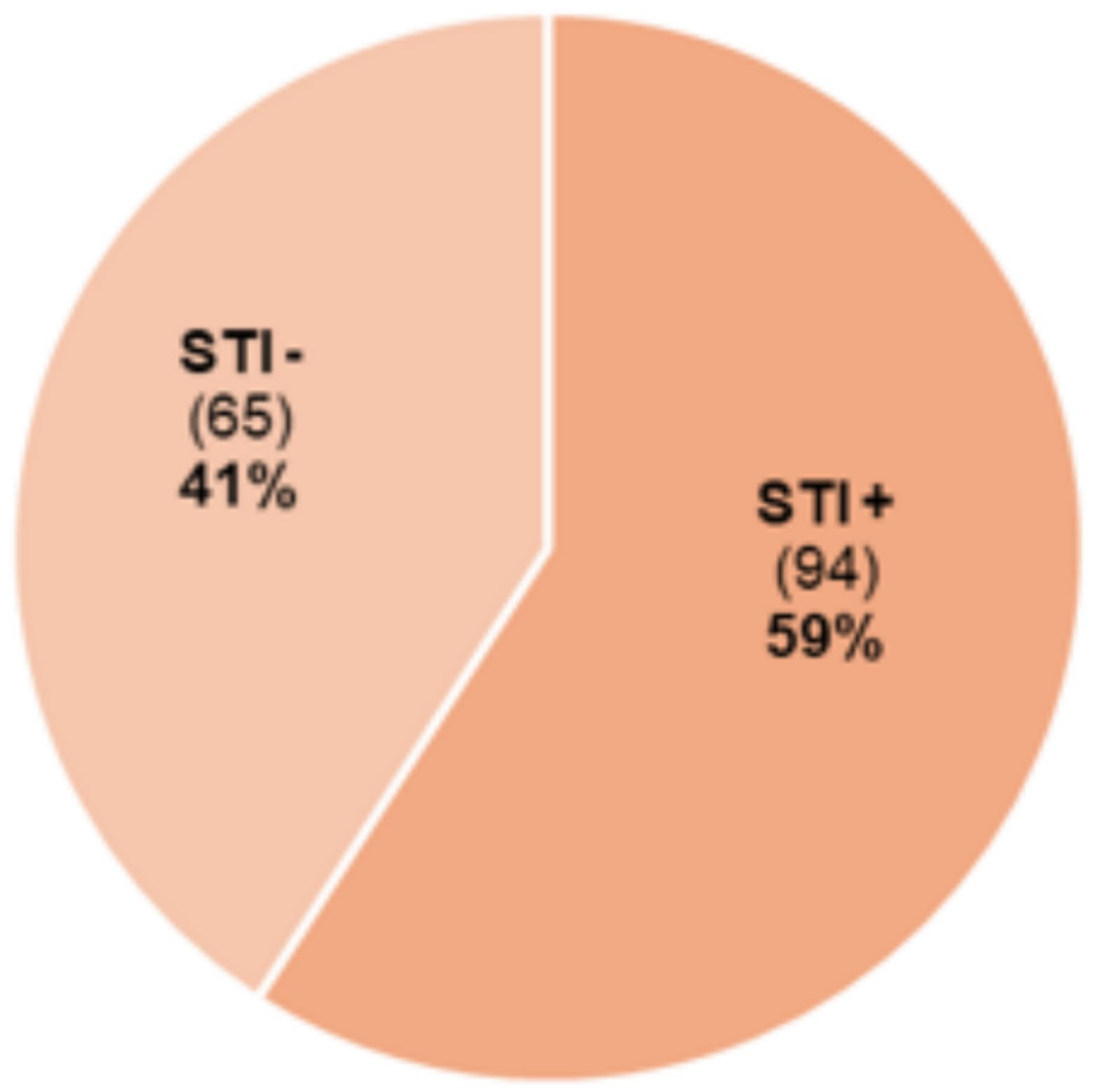
Prevalence of non-HPV sexually transmitted infections (STI) among patients with cervical dysplasia.

Education levels varied among participants, with 42% having completed high school, 15% not completing elementary school, and only 9.2% holding a university degree. When asked about their sexual history, 21.4% reported having up to two lifetime sexual partners, 28.9% had between two and five, 25.2% had between 5 and 10, and 21.4% had more than 10 partners. A total of 94 patients with dysplasia (59.1%; we excluded condyloma) had co-infections with non-HPV STIs, while 65 (40.8%) did not (Figure 2).

Overall, the most frequently detected pathogen was *U. urealyticum* [when isolated in 53% (50/94) and when associated in 72.1% (67/94), followed by *M. hominis* (10.6%) and *C. trachomatis* (7.4%) (Table 1)].

**Table 1:** Prevalence of STI (in absolute numbers and percentage) found in different histopathological types (HT).

HT	UU	UU + MH	UU + CT	UU + CT + NG	MG	MH	CT	NG	CT + NG	CT + NG + TV	TV	Total
CIN I	19 (79,1%)	2 (8,3%)	0	0	2 (8,3%)	0	1 (4,1%)	0	0	0	0	24 (100%)
CIN II/III	27 (44,2%)	10 (16,3%)	1 (1,6%)	1 (1,6%)	0	8 (13,1%)	6 (10%)	0	4 (6,5%)	3 (5%)	1 (1,6%)	61 (99,9%)
VIN	2 (50%)	2 (50%)	0	0	0	0	0	0	0	0	0	4 (100%)
SCC	1 (50%)	1 (50%)	0	0	0	0	0	0	0	0	0	2 (100%)
Adeno *In Situ*	1 (50%)	1 (50%)	0	0	0	0	0	0	0	0	0	2 (100%)
Condyloma	0	0	0	1 (100%)	0	0	0	0	0	0	0	1 (100%)
Total	50 (53%)	16 (17%)	1 (1%)	2 (2%)	2 (2%)	8 (8,5%)	7 (7,4%)	0	4 (4,2%)	3 (3%)	1 (1%)	94 (99,1%)

UU, *Ureaplasma urealyticum*; MH, *Mycoplasma hominis*; MG, *Mycoplasma genitalium*; CT, *Chlamydia trachomatis*; NG, *Neisseria gonorrhoeae*; TV, *Trichomonas vaginalis*; CIN I, Cervical Intraepithelial Neoplasia I; CIN II/III, Cervical Intraepithelial Neoplasia II/III; VIN, Vulvar Intraepithelial Neoplasia; SCC, Squamous Cell Carcinoma; ADENO IN SITU, Adenocarcinoma in situ; CONDYLOMA, Condyloma acuminatum.

Among patients with low- and high-grade lesions, non-HPV STIs were present in more than 50% of cases. In patients with VIN, adenocarcinoma *in situ,* and squamous cell carcinoma, 100% tested positive for non-HPV STIs. In the CIN I group, 79.1% of patients tested positive for *U. urealyticum,* while a smaller number had infections with *M. genitalium, C. trachomatis,* or multiple pathogens. In the CIN II/III group, *U. urealyticum* was the most frequently detected pathogen, present either alone (44.2%) or in combination with other STIs (65.2%), reaching a 64.8% positivity rate. *Mycoplasma hominis* was the second most common, detected alone in 13.1% of cases and in association with *U. urealyticum* in 29.5%. *Chlamydia trachomatis* was detected in 7.4% of cases alone and in 7.2% in combination with other pathogens, totaling 15% positivity. In VIN, adenocarcinoma *in situ*, and squamous cell carcinoma cases, *U. urealyticum* was detected in all patients, with some presenting *U. urealyticum* alone and the others, *U. urealyticum* associated with *M. hominis* and /or associated with *Mycoplasma genitalium* (Table 1).

## 4 Discussion

Cervical cancer remains highly prevalent in LMICs, but there is strong scientific evidence demonstrating that its prevention is both feasible and effective. This is primarily due to several factors: it is preceded by a progressive cervical dysplasia, the cervix is easily accessible for routine screening (including cytology, colposcopy, biopsy, treatment, and observation), it is associated with modifiable local factors, it is not typically fatal when detected early, and it primarily affects young, sexually active women, who can be targeted in screening programs (24, 25).

This neoplasia is strongly linked to HPV infection, the most widespread STI globally. However, fewer than 2% of HPV-infected individuals will develop cervical cancer, as most women are able to clear the infection through their immune response (7, 8, 10, 11, 24). Local factors play a key role in HPV persistence, and several studies have suggested that co-infection with non-HPV STIs contributes to viral persistence and disease progression (2, 7, 8, 10, 11, 24). Women with co-infections have lower HPV clearance rates, greater persistence, more cytopathological abnormalities, and are twice as likely to develop cervical dysplasia (26).

In addition to their potential involvement in cervical carcinogenesis, non-HPV STIs pose significant health risks for young, reproductive-aged women, leading to pelvic inflammatory disease, chronic pelvic pain, infertility, and recurrent ectopic pregnancy (2, 4, 10, 24, 27). During pregnancy, these infections can have severe maternal and neonatal consequences, including fetal loss, preterm labor, premature rupture of membranes, chorioamnionitis, low birth weight, neonatal pneumonia and conjunctivitis, puerperal endometritis, and neonatal sepsis. Despite these risks, the Brazilian Ministry of Health mandates screening for cervical cancer but does not include STI screening as part of its national program (21, 22). As a result, there is no comprehensive data on the true prevalence of these infections in Brazil. In contrast, the U.S.

Preventive Services Task Force recommends universal *C. trachomatis* screening for all sexually active women aged 25 and older, 29 citing its benefits as significantly outweighing potential harms. In Brazil, cervical cytology (Pap smear) is routinely performed, and patients with abnormal results are referred to specialized centers for diagnostic confirmation and treatment (21). In our study, we included 159 patients diagnosed with CIN at a referral hospital, aiming to evaluate the prevalence of non-HPV STIs, specifically *Chlamydia trachomatis*, *N. gonorrhoeae, Ureaplasma urealyticum, M. hominis, M. genitalium, and T. vaginalis.* After diagnosis, treatment was prescribed for both the patients and their sexual partners.

Most of our patients presented with high-grade CIN (64.8%), which is likely related to the fact that we are a tertiary referral center. Regarding demographic factors, the mean age was 41.8 years, and most patients had a low income (≤2 minimum wages), had multiple sexual partners (>4), initiated sexual activity early (≤16 years), and self-identified as white. While most patients were non-smokers, 24.5% reported smoking. Several studies have identified age, number of sexual partners, early sexual debut, and smoking as risk factors for HPV persistence, though some researchers have not replicated these findings, making them a topic of ongoing debate (27).

Most of our patients were over 35 years old, so we did not observe a higher prevalence of STIs in younger women, as reported in studies from Peru. However, we did find similarities in sexual behavior, as 78.6% of our sample reported more than three lifetime sexual partners, which aligns with previous studies identifying this as a risk factor (6). Socioeconomic improvements and increased education levels have been associated with greater female empowerment, leading to delayed sexual debut and better reproductive choices (28).

Bose et al. reported that higher education and sexual initiation after age 21 were independently associated with a lower prevalence of CIN and cervical cancer, with a 37% reduction in high-grade CIN among women with a high school education or higher (28). In our study, most participants were low-income, 89% initiated sexual activity before age (21), and only 9.2% had a university degree, indicating a strong correlation between lower education, socioeconomic vulnerability, and increased CIN risk.

Although there are conflicting opinions, smoking is widely regarded as a cofactor in HPV-related cervical carcinogenesis. Its effects appear to be dose-dependent and linked to smoking duration, with studies suggesting that cessation eliminates this risk over time. Nicotine and its toxic metabolites have been shown to damage cervical mucus, reduce immune cell activity (particularly Langerhans cells) and impair the immune response against HPV (29).

Kayar et al. (29) reported a higher incidence of cervical dysplasia among smokers compared to non-smokers (28% vs. 11.8%), while Du et al. (30) found that passive smoking also increased risk. In our study, 24.5% of patients were smokers, with the highest rates among patients with VIN (50%). Given the multiple harms associated with smoking, patients were counseled on smoking cessation and referred for professional support. Even among asymptomatic patients, we found a high prevalence of non-HPV STIs in women with CIN (59%), with *U. urealyticum* (44%), *M. hominis* (16.6%), and *C. trachomatis* (10.1%) being the most frequently detected pathogens. *Ureaplasma urealyticum* was the most prevalent across all degrees of cervical dysplasia, and 15% of patients had co-infection with two or more pathogens.

*Chlamydia trachomatis*, whose association with CIN and cervical cancer is well established, has been reported with varying prevalence rates across different LMICs (4, 7, 9, 17, 27, 31, 32). Our 10.1% prevalence was similar to a study conducted in Carazinho, Brazil (9.5%), but lower than findings from Belo Horizonte, Brazil (27.8%), Bogotá (28%), among others. Interestingly, Spain reported a lower prevalence (2.8%), suggesting potential regional and socioeconomic influences on STI epidemiology (33).

*Ureaplasma urealyticum, M. hominis,* and *M. genitalium* belong to the *Mycoplasmataceae* family, which comprises opportunistic genital tract pathogens that often cause silent infections and have been linked to HPV persistence (10, 20). In our study, 66.1% of patients tested positive for the *Mycoplasmataceae.* Other studies have reported prevalence rates ranging from 1.8 to 84%, with variations likely due to differences in population, diagnostic methods, and study settings (4, 32, 34). The pathogenic mechanisms by which the *Mycoplasmataceae* contributes to cervical dysplasia remain unclear, but evidence suggests that chronic inflammation, immune dysfunction, free radical generation, and epithelial barrier disruption facilitate HPV persistence and oncogenesis (14, 15, 20).

*Neisseria gonorrhoeae* was detected in 3.8% of patients, while *T. vaginalis* was the least prevalent STI (1.2%), contrasting with higher rates reported in other studies (14.4–24.8%) (2, 10).

Several studies have demonstrated high STI prevalence in asymptomatic HPV-infected women, supporting the need for routine STI screening in CIN patients (4, 10, 14, 15, 19, 35, 36, 37). Some authors propose that self-collection kits for HPV and STI testing could be a valuable tool, especially for women referred for colposcopy at specialized centers (34). Expanding STI screening programs would not only improve early diagnosis and treatment but also enhance awareness and prevention strategies to curb disease transmission (26).

Our findings align with those from other LMICs, reinforcing the global relevance of STI co-infection in CIN patients. Despite regional variations, cervical cancer remains a major public health concern, affecting young women and their families daily.

This study has some limitations. As a tertiary referral center, our study lacks a control group of women without CIN, which limits direct comparisons. Additionally, molecular HPV DNA testing was not performed, though we mitigated this limitation by including only histopathologically confirmed CIN cases. Given Brazil’s regional disparities, generalizing our findings to other states may be challenging.

## 5 Conclusion

This study revealed a strong association between non-HPV STIs, particularly the *Mycoplasmataceae*, and CIN. Further longitudinal studies are needed to clarify the causal role of STIs in cervical dysplasia, but in the meantime, STI screening should be integrated into cervical cancer prevention strategies to enhance early detection, treatment, and overall public health impact.

## Data Availability

The original contributions presented in the study are included in the article/supplementary material, further inquiries can be directed to the corresponding author.
